# Supplementary material for: Critical Reappraisal of Methods for Measuring Urine Saturation with Calcium Salts
Source: Molecules. 2021 May 25;26(11):3149. doi: 10.3390/molecules26113149 (PMC8197498; doi:10.3390/molecules26113149)
Supplement: Supplementary file 1 [file molecules-26-03149-s001.zip › molecules-1230994-supplementary.pdf]

Supplementary Materials

Critical Reappraisal of Methods for Measuring Urine Saturation with Calcium Salts

Silvia Berto [1,*], Martino Marangella [2], Concetta De Stefano [3], Demetrio Milea [3] and Pier Giuseppe Daniele [1,†]

[1] Dipartimento di Chimica, Università di Torino, via P. Giuria 7, 10125 Torino, Italy; pierodan2015@gmail.com

[2] Fondazione Scientifica Mauriziana-Onlus, via Magellano 1, Torino, Italy; mmarangella@alice.it

[3] Dipartimento di Scienze Chimiche, Biologiche, Farmaceutiche ed Ambientali, Università degli Studi di Messina, CHIBIOFARAM, Viale Ferdinando Stagno d'Alcontres 31, 98166 Messina, Italy; cdestefano@unime.it (C.D.S.); dmilea@unime.it (D.M.)

* Correspondence: silvia.berto@unito.it

1. Calcium complexes in urinary conditions

Table 1S - pH and mmolar concentrations of the components of the urine samples.

		**Urine components amounts** (mmol L^{-1})									
**urine**	**pH**	**Ca^{2+}**	**Mg^{2+}**	**Na^{+}**	**K^{+}**	**NH_4^{+}**	**Cl^{-}**	**PO_4^{3-}**	**SO_4^{2-}**	**cit^{3-} [1]**	**ox^{2-} [2]**
A	7.50	1.23	1.67	65.9	33.2	13.3	68.2	6.91	3.00	1.88	0.17
B	7.24	2.28	1.50	59.0	33.0	16.6	66.0	25.8	19.2	0.85	0.36
C	7.06	1.23	0.80	23.1	24.0	9.14	31.1	10.5	3.26	0.27	0.08
D	6.84	1.78	1.54	74.1	21.8	14.4	91.2	17.1	16.6	1.64	0.30
E	6.57	2.90	2.95	56.8	29.1	10.8	63.2	8.2	6.3	0.75	0.24

[1] cit^{3-} = citrate anion
[2] ox^{2-} = oxalate anion

Table 2S - Overall (log*β*) and partial (log*K*) formation constants of the species considered to model real urine samples.

Species	I = 0.16 mol L^{-1}, t = 37°C		reference
	log*K*	log*β*	
HPO_4^{2-}	11.64	11.64	[1]
$H_2PO_4^-$	6.83	18.47	[1]
H_3PO_4	2.03	20.51	[1]
$Hcit^{2-}$ [1]	5.80	5.80	[2]
H_2cit^-	4.31	10.11	[2]
H_3cit	2.86	12.97	[2]
Hox^- [2]	3.98	3.98	[3]
CaHcit	2.03	7.83	[4]
$Cacit^-$	3.49	3.49	[4]
$[Ca(PO_4)cit]^{4-}$	8.3±0.8	8.3±0.8	This work [3]
MgHcit	1.74	7.54	[4]
$Mgcit^-$	3.54	3.54	[4]
$NaHcit^-$	0.58	6.38	[5]
$Nacit^{2-}$	0.98	0.98	[5]
Na_2cit^-	1.40	1.40	[5]
$Kcit^{2-}$	0.56	0.56	[2]
NH_4Hcit^-	0.47	6.27	[6]
NH_4cit^{2-}	0.95	0.95	[6]
Caox	2.36	2.36	[7]
Mgox	2.67	2.67	[7]
$Naox^-$	0.51	0.51	[3]
Kox^-	0.41	0.41	[3]
NH_4ox^-	0.65	0.65	[6]
$CaH_2PO_4^+$	1.09	19.56	[8]
$CaHPO_4$	1.97	13.61	[8]
$CaPO_4^-$	6.08±0.02	6.08±0.02	This work [3]
$MgH_2PO_4^+$	1.20	19.67	[8]
$MgHPO_4$	2.11	13.75	[8]
$MgPO_4^-$	3.4	3.4	[9]
$NaHPO_4^-$	0.77	12.41	[1]
$NaPO_4^{2-}$	0.95	0.95	[1]
$KHPO_4^-$	0.58	12.22	[1]
KPO_4^{2-}	0.85	0.85	[1]
$NH_4HPO_4^-$	0.84	0.84	[6]
$CaSO_4$	1.60	1.60	[7]
$MgSO_4$	1.65	1.65	[7]
$NaSO_4^-$	0.40	0.40	[7]
KSO_4^-	0.54	0.54	[7]
$NH_4SO_4^-$	0.92	0.92	[6]

[1] cit = citrate anion

[2] ox = oxalate anion

[3] Values extrapolated from those at I = 0.1 M upon application of an Extended Debye–Hückel equation [10]

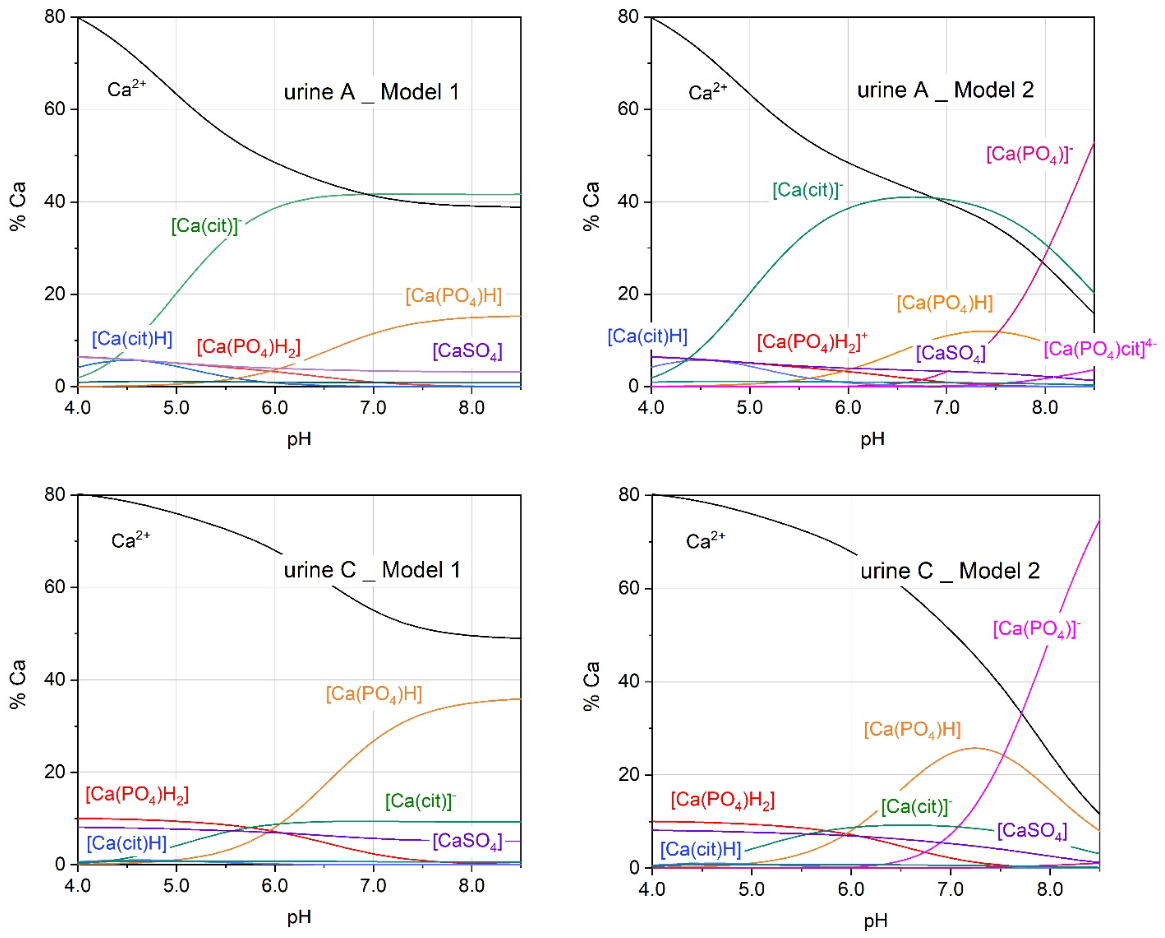

Figure 1S - Speciation plots for the urine A and C obtained with Model 1 (species $[CaPO_4]^-$ and $[Ca(PO4)cit]^{4-}$ excluded) and Model 2 (species $[CaPO_4]^-$ and $[Ca(PO_4)cit]^{4-}$ included).

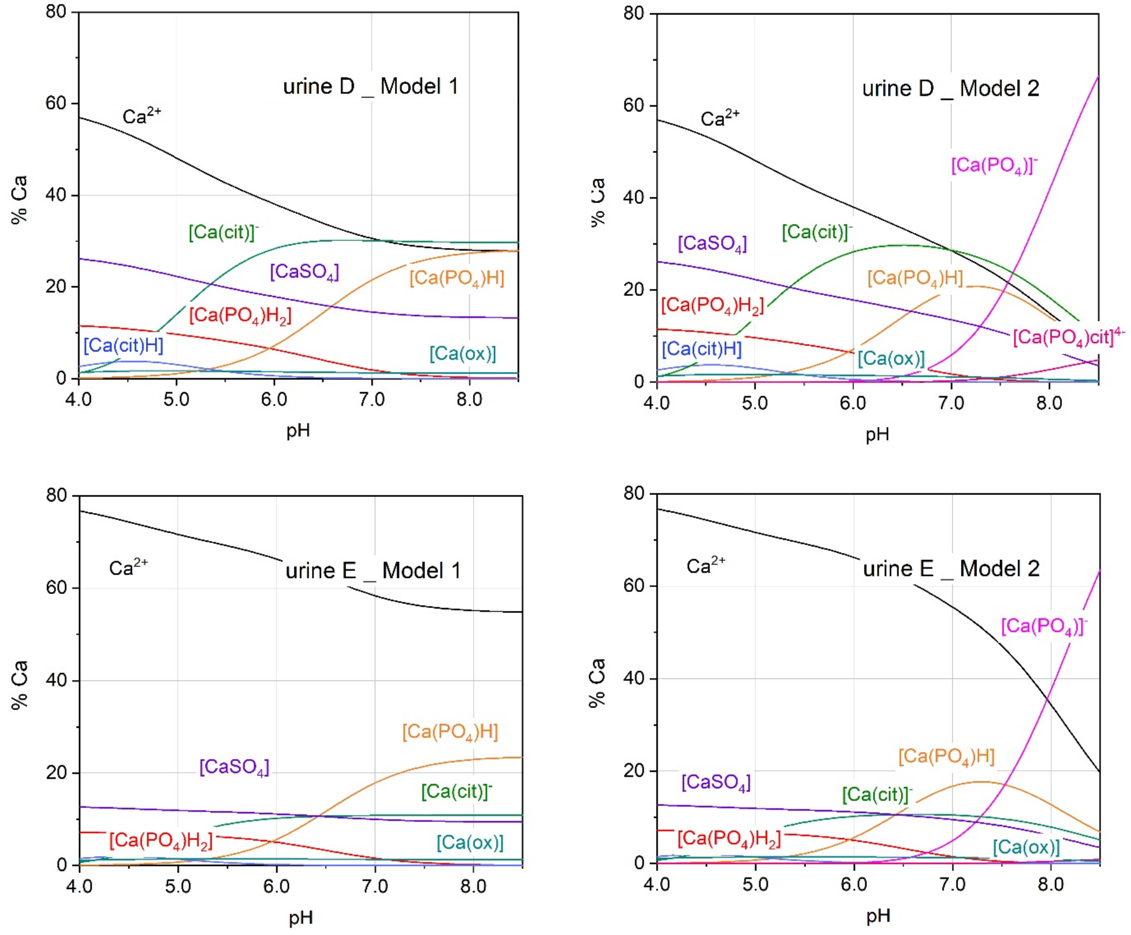

Figure 2S - Speciation plots for the urine D and E obtained with Model 1 (species $[CaPO_4]^-$ and $[Ca(PO4)cit]^{4-}$ excluded) and Model 2 (species $[CaPO_4]^-$ and $[Ca(PO_4)cit]^{4-}$ included).

2. Saturation level calculation.

Saturation level was represented by the values of β and calculated as previously described [11]:

$$\beta_{Caox} = \frac{[Ca^{2+}][ox^{2-}]}{K_{sp,Caox}}$$

$$\beta_{CaHPO_4} = \frac{[Ca^{2+}][HPO_4^{2-}]}{K_{sp,CaHPO_4}}$$

where $[Ca^{2+}]$, $[ox^{2-}]$ (ox^{-2} = oxalate anion) and $[HPO_4^{2-}]$ are the concentrations of the species at chemical equilibrium estimated by the application of the chemical model. $K_{sp,Caox}$ and $K_{sp,CaHPO_4}$ are the solubility products of the solid species Caox and $CaHPO_4$ assessed at the ionic strength calculated with the model application, at the real pH of the sample. The values of $K_{sp,Caox}$ and $K_{sp,CaHPO_4}$ were assessed at different ionic strength as reported in the ref. [10]. Both species concentrations and ionic strengths refer to the pH value of the real sample. If $\beta > 1$, the urine is supersaturated. This does not mean necessarily the formation of stones.

References

1. Daniele PG, De Robertis A, De Stefano C, Gianguzza A, Sammartano S. Salt effect on the protonation of ortho-phosphate between 10 and 50°C in aqueous solution. A complex formation model. *Journal of Solution Chemistry* 1991, 20(5), 495-515.
2. Daniele PG, Rigano C, Sammartano S. The formation of proton and alkali-metal complexes with ligands of biological interest in aqueous solution. Potentiometric study of the H^+-K^+-citrate system at 37°C and 0.03 < I < 1.0. *Annali di Chimica* 1980, 72(1), 119-130.
3. Daniele PG, Rigano C, Sammartano S. The formation of proton and alkali-metal complexes with ligands of biological interest in aqueous solution. Thermodynamics of H^+, Na^+ and K^+ -oxalate complexes. *Thermochimica Acta* 1981, 46, 103-116.
4. Amico P, Daniele PG, Rigano C, Sammartano S. Stability of calcium- and magnesium-citrate complexes in aqueous solution. *Annali di Chimica* 1982, 72, 1-24.
5. Daniele PG, De Robertis A, De Stefano C, Gianguzza A, Sammartano S. Studies on Polyfunctional O-Ligands. Formation Thermodynamics of Simple and Mixed Alkali Metal Complexes with Citrate at Different Ionic Strengths in Aqueous Solution. *Journal of Chemical Research* (S) 1990, 300-301.
6. Amico P, Daniele PG, Rigano C, Sammartano S. Formation and stability of ammonium-sulphate, phosphate, oxalate and citrate complexes in aqueous solution. *Annali di Chimica* 1981, 71, 659-667.
7. Daniele PG, Marangella M. Ionic equilibria in urine: a computer model system improved by accurate stability constant values. *Annali di Chimica* 1982, 72, 25-38.
8. Daniele PG, Rigano C, Sammartano S. Formation and stability of calcium- and magnesium-phosphate complexes in aqueous solution at 37°C. A potentiometric investigation by glass and calcium ion-selective electrodes in the ionic strength range 0.03 < I < 0.5. *Annali di Chimica* 1982, 72, 341-353.

9. Childs CW. A potentiometric study of equilibria in aqueous divalent metal orthophosphate solutions. *Inorganic Chemistry* 1970, 9(11), 2465-2469.
10. Casale A, Daniele PG, De Robertis A, Sammartano S. Ionic strength dependence of formation constants. XI. An analysis of literature data on carboxylate ligand complexes. *Annali di Chimica* 1988, 78, 249-260.
11. Daniele PG, Sonego S, Ronzani M, Marangella M. Ionic strength dependence of formation constants. Part 8. Solubility of calcium oxalate monohydrate and calcium hydrogenphosphate dihydrate in aqueous solution, at 37°C and different ionic strength. *Annali di Chimica* 1985, 75, 245-250.
